# Treatment of false‐negative metastatic lymph nodes by a lymphatic drug delivery system with 5‐fluorouracil

**DOI:** 10.1002/cam4.2125

**Published:** 2019-04-03

**Authors:** Honoka Fujii, Sachiko Horie, Ariunbuyan Sukhbaatar, Radhika Mishra, Maya Sakamoto, Shiro Mori, Tetsuya Kodama

**Affiliations:** ^1^ Laboratory of Biomedical Engineering for Cancer Graduate School of Biomedical Engineering Tohoku University Aoba, Sendai Miyagi Japan; ^2^ Biomedical Engineering Cancer Research Center Graduate School of Biomedical Engineering Tohoku University Aoba, Sendai Miyagi Japan; ^3^ Department of Oral and Maxillofacial Surgery Tohoku University Aoba, Sendai Miyagi Japan; ^4^ Department of Biological Sciences Indian Institute of Science Education and Research Bhopal Bhopal Madhya Pradesh India; ^5^ Department of Oral Diagnosis Tohoku University Hospital Aoba, Sendai Miyagi Japan; ^6^ Department of Oral and Maxillofacial Surgery Tohoku University Hospital Aoba, Sendai Miyagi Japan

**Keywords:** false‐negative, fluorouracil (5‐FU), lymph node, lymphatic drug delivery system, metastasis, mouse

## Abstract

Metastatic lymph nodes (LNs) may be the origin of systemic metastases. It will be important to develop a strategy that prevents systemic metastasis by treating these LNs at an early stage. False‐negative metastatic LNs, which are found during the early stage of metastasis development, are those that contain tumor cells but have a size and shape similar to LNs that do not host tumor cells. Here, we show that 5‐fluorouracil (5‐FU), delivered by means of a novel lymphatic drug delivery system (LDDS), can treat LNs with false‐negative metastases in a mouse model. The effects of 5‐FU on four cell lines were investigated using in vitro cytotoxicity and cell survival assays. The therapeutic effects of LDDS‐administered 5‐FU on false‐negative metastatic LNs were evaluated using bioluminescence imaging, high‐frequency ultrasound (US), and histology in MHX10/Mo‐*lpr*/*lpr* mice. These experimental animals develop LNs that are similar in size to human LNs. We found that all cell lines showed sensitivity to 5‐FU in the in vitro assays. Furthermore, a concentration‐dependent effect of 5‐FU to inhibit tumor growth was observed in tumor cells with low invasive growth characteristics, although a significant reduction in metastatic LN volume was not detected in MHX10/Mo‐*lpr*/*lpr* mice. Adverse effects of 5‐FU were not detected. 5‐Fluorouracil administration with a LDDS is an effective treatment method for false‐negative metastatic LNs. We anticipate that the delivery of anticancer drugs by a LDDS will be of great benefit in the prevention and treatment of cancer metastasis via LNs.

## 1 INTRODUCTION

Lymph node (LN) metastasis in patients with cancer is considered to be an important predictor of poor prognosis. There has been much debate about how tumor cells spread to distant organs hematogenously via LNs.^1^, ^2^, ^3^, ^4^ Recently, several papers have reported that metastatic LNs could be the origin of systemic metastases.^5^, ^6^, ^7^ Brown et al^5^ determined that high endothelial venules play an important role in hematogenous metastasis, and Pereira et al^7^ concluded that tumor cells invade local vessels in LNs and that this is followed by hematogenous metastasis. Kodama et al^6^ demonstrated that the initiation of hematogenous metastasis is tumor cell invasion from the marginal sinus of a LN into the extranodal veins, and the authors advocated the theory of LN‐mediated hematogenous metastasis.^6^, ^8^, ^9^ Several clinical trials have indicated that surgical excision of tumor‐draining LNs has no effect on the long‐term survival of patients with cancer.^10^, ^11^, ^12^ These findings suggest that tumor cells are seeded throughout the body at the stage where micrometastasis exists in the LNs, and as a result, surgical dissection does not affect the long‐term survival of cancer patients; this result would support the validity of the LN‐mediated hematogenous metastasis theory.

Although the theory of LN‐mediated hematogenous metastasis needs confirmation in a clinical setting, it is clear that a new strategy is required that prevents systemic metastasis by treating metastatic LNs at the early stage of metastasis. Since LNs have a rich vascular network,^13^ tumor cells can develop in them without tumor angiogenesis.^14^, ^15^ Thus, hematogenous delivery of macromolecular drugs based on the enhanced permeability and retention effect^16^ and normalization of the circulation (ie, improvement of the flow and functions of the vasculature)^17^ would not be effective in the treatment of metastatic LNs. In addition, the lymphatic system preferentially takes up large‐sized particles and high molecular weight substances. Particles 10‐100 nm in size are reabsorbed from the interstitial space by the lymphatic system,^18^, ^19^ while those < 10 nm in size are primarily reabsorbed by blood vessels. The efficiency of reabsorption into the lymphatic system correlates positively with molecular weight, and substances with a molecular weight above 16 000 g/mol are mainly taken up by the lymphatic system.^20^, ^21^ Since anticancer drugs are usually small molecules, generally they are poorly delivered to the lymphatic system after systemic administration.

For the above reasons, the use of a LDDS to deliver anticancer drugs efficiently to false‐negative metastatic LNs during the early stage of metastasis has been proposed.^22^ False‐negative metastatic LNs are those that contain tumor cells but, due to the early stage of tumor development, have a size and shape similar to those of LNs not hosting tumor cells. The LDDS is a new approach to treat or prevent LN micrometastasis, and we have previously demonstrated this novel therapeutic concept.^23^ The LDDS operates by injecting drugs into sentinel (upstream) LNs in order to deliver them to downstream metastatic LNs in the lymphatic network and maintain high concentrations of drug within the targeted LNs.

The aim of this investigation was to examine the therapeutic effects of 5‐FU administered through a LDDS on false‐negative metastatic LNs. 5‐Fluorouracil has been widely used to treat LN metastasis in breast and gastric cancer. First, the antitumor effects of 5‐FU were assessed in four different cell lines using in vivo cytotoxicity and cell survival assays. Next, mouse models of LN metastasis (generated using two tumor cell lines with different growth characteristics) were utilized to evaluate the therapeutic effects of LDDS‐administered 5‐FU on false‐negative metastatic LNs. It was found that by using a LDDS, the administration of 5‐FU is an effective treatment for LNs with false‐negative metastases.

## 2 METHODS

The Tohoku University Institutional Animal Care and Use Committee approved the in vivo study protocols.

### 2.1 Culture of cells

Cultured cells used in the experiments were MRL/MpTn‐*gld*/*gld* mouse malignant KM‐Luc/GFP cells that stably expressed a fusion of the enhanced‐green fluorescent protein and luciferase genes were maintained in culture,^24^ as were BALB/cCrgl mouse mammary carcinoma cells (EMT6‐Luc) that stably expressed the firefly luciferase gene,^25^ C3H/He mouse Dunn osteosarcoma cells (LM8‐Luc) stably expressing the firefly luciferase gene,^26^ and C3H/He mouse mammary carcinoma cells (FM3A‐Luc) stably expressing the Luc gene.^27^ KM‐Luc/GFP, EMT6‐Luc and LM8 cells were cultured in Dulbecco's modified Eagle's medium (DMEM; Sigma‐Aldrich, St Louis, MO) supplemented with 10% heat‐inactivated fetal bovine serum (FBS; HyClone Laboratories Inc., UT, US) and 1% l‐glutamine–penicillin‐streptomycin (Sigma‐Aldrich). FM3A‐Luc cells were maintained in RPMI‐1640 medium supplemented with 10% (v/v) FBS (HyClone Laboratories Inc.), 1% (v/v) l‐glutamine–penicillin‐streptomycin and 1 mg/mL G418 (Wako Pure Chemical Industries, Osaka, Japan). Cell lines were incubated at 37°C in an atmosphere of 5% CO_2_ and 95% air until 80% confluence was achieved. A lack of *Mycoplasma* contamination was confirmed for all in vivo experiments on the inoculation day using a MycoAlert *Mycoplasma* Detection Kit (Lonza Rockland, ME, US).

### 2.2 In vitro cytotoxicity and cell survival assays

The cytotoxic effects of 5‐FU on KM‐Luc/GFP cells (n* *=* *8) and FM3A‐Luc cells (n* *=* *8) in 96‐well plates were measured using a lactate dehydrogenase (LDH) assay according to the protocol provided by the kit manufacturer (LDH‐cytotoxicity kit; Wako Pure Chemical Industries). The cytotoxicity ratio was calculated thus:

(1) $$\mathrm{Cytotoxicity}\;\mathrm{ratio}\;\left(\%\right)=\frac{S-N}{P-N}\times 100$$

where *S* is the mean absorbance of the wells to which 5‐FU was added, *N* the average absorbance of negative control wells, and *P* the average absorbance of positive control wells. The MTT assay was carried out for KM‐Luc/GFP (n* *=* *12), FM3A‐Luc (n =* *12), EMT6‐Luc (n* *= 7), and LM8‐Luc (n =* *8) cells in 96‐well plates, as previously described.^28^ The cell survival ratio was determined by the following equation:

(2) $$\mathrm{Cell}\;\mathrm{survival}\;\mathrm{ratio}\;\left(\%\right)=\frac{A_{x}}{A_{0}}\times 100$$

where $A_{x}$ is the average absorbance of the wells to which 5‐FU was added and $A_{0}$ the average absorbance of the wells without 5‐FU.

### 2.3 Mice

MHX10/Mo‐*lpr*/*lpr* (MXH10/Mo/lpr) mice (aged 16‐18 weeks)^27^ were bred under specific pathogen‐free conditions in the Animal Research Institute, Tohoku University. MXH10/Mo/lpr mice are exceptional in that their peripheral LNs develop to 10 mm in size at 2.5 to 3 months, and they do not exhibit autoimmune disease. In the present study, we used the term “subiliac LN” instead of “inguinal LN”^29^ to assign the correct anatomical locations and nomenclatures to murine LNs.

### 2.4 Induction of metastasis in the proper axillary LN (PALN) by injection of tumor cells into the subiliac LN (SiLN)

Mice were anesthetized (2% isoflurane in O_2_), and the unilateral SiLN injected (under US guidance) with KM‐Luc/GFP cells (3.3 × 10^5^ cells/mL) or FM3A‐Luc cells (3.3 × 10^5^ cells/mL) suspended in 10 μL phosphate‐buffered saline plus 20 μL of 400 mg/mL Matrigel (Collaborative Biomedical Products, Bedford, MA). Tumor cells were injected into the SiLN to induce metastasis in the PALN. The day of tumor cell inoculation was defined as day 0.^23^ Tumor growth in the PALN was assessed with an in vivo bioluminescence imaging system (IVIS; PerkinElmer, Waltham, MA, US). Luciferase activity was measured on day 3, day 6, and day 9 for KM‐Luc/GFP cells. Metastasis of the PALN was considered to have occurred when the luciferase activity exceeded the background level in controls (2 × 10^5^ photons/s) on day 6. The day on which PALN metastasis was confirmed was defined as day 0^T^. For FM3A‐Luc cells, PALN tumor growth was assessed on day 7, day 14, and every 3 days after day 14. PALN metastasis was considered to have occurred when the luciferase activity exceeded the background level in controls (1 × 10^6^ photons/s). The day on which PALN metastasis was confirmed was defined as day –1^T^.

### 2.5 Treatment of the metastatic PALN with 5‐FU

Mice with confirmed metastasis of KM‐Luc/GFP cells to the PALN were randomly divided into four groups based on the concentration of 5‐FU administered: control (saline alone, ie, 0 μg/g; n* *=* *4), 0.1 μg/g (n =* *4), 1 μg/g (n =* *4), and 10 μg/g (n =* *4). Mice with confirmed metastasis of FM3A‐Luc cells to the PALN were randomly divided into five groups thus: control (saline alone, ie, 0 μg/g; n =* *6), 0.1 μg/g (n =* *5), 1 μg/g (n =* *5), 10 μg/g (n =* *5), and 100 μg/g (n* *=* *5). Each mouse was deeply anesthetized with 2% isoflurane (Abbott Japan, Chiba, Japan) in O_2_, and 120 μL of the appropriate solution was injected into the accessory axillary LN (AALN). Injection of 5‐FU solution into the AALN was carried out with a 27‐G butterfly needle at an injection speed of 10 μL/min using a syringe pump (Legato100; KD Scientific, Holliston, MA, US) under the guidance of a high‐frequency ultrasound imaging system (VEVO770; VisualSonics, Toronto, Canada) with a 25‐MHz transducer (RMV‐710B; axial resolution, 70 μm; focal length, 15 mm; VisualSonics) on day 0^T^.

### 2.6 Measurement of SiLN and PALN volumes

SiLN and PALN volumes were measured using a high‐frequency ultrasound imaging system (VEVO770; Visual Sonics) with a 25‐MHz transducer (RMV‐710B; Visual Sonics) on day 0 (ie, ‐6^T^), day 6 (ie, 0^T^), and day 9 (ie, 3^T^) for KM‐Luc/GFP cells and on day 0, day 0^T^, and day 9^T^ for FM3A‐Luc cells.

### 2.7 Ex vivo measurement of tumor growth

The PALN was harvested on day 3^T^ for KM‐Luc/GFP cells and on day 9^T^ for FM3A‐Luc cells. Luciferase activity in the samples (in six‐well plates) was measured with the IVIS (0.3 mg/mL luciferin). Luciferase activity was evaluated on both sides of each sample to obtain the average luciferase activity.

### 2.8 Blood biochemistry to assess renal/hepatic toxicity of 5‐FU

Mice were assigned into five groups based on the tumor cell line and 5‐FU concentration used thus: KM‐Luc/GFP, 0 μg/g (n =* *4); KM‐Luc/GFP, 10 μg/g (n =* *4); FM3A‐Luc, 0 μg/g (n =* *6); FM3A‐Luc, 10 μg/g (n =* *5); and FM3A‐Luc, 100 μg/g (n =* *5). Blood was drawn from the left ventricle on day 3^T^ (KM‐Luc/GFP) or day 9^T^ (FM3A‐Luc), and plasma was obtained by centrifugation at 13 000 *g* for 10 seconds. Hepatic and renal functions were evaluated from plasma measurements of total bilirubin (T‐BIL), alanine aminotransferase (ALT), aspartate aminotransferase (AST), creatinine (Cre), and blood urea nitrogen (BUN) (Oriental Yeast, Tokyo, Japan).

### 2.9 Weight measurement

To evaluate any acute toxic effects of 5‐FU, the body weights of mice were measured and recorded. For mice inoculated with KM‐Luc/GFP cells, body weights were measured on days 0, 3, 6 (0^T^), and 9 (3^T^). For mice inoculated with FM3A‐Luc cells, body weights were measured on days 0, ‐1^T^, 0^T^, 3^T^, 6^T^, and 9^T^.

### 2.10 Histological analysis

Tissues (PALN, AALN, lung, liver, and kidney) were excised on day 3^T^ (KM‐Luc/GFP) or day 9^T^ (FM3A‐Luc), fixed overnight in 10% formalin at 4°C, dehydrated, embedded in paraffin, serially sectioned (3‐4 μm), and either stained with hematoxylin and eosin (H&E) or immunostained for LYVE‐1‐positive and CD31‐positive cells (Discovery XT Automated Staining Processor; Ventana Medical Systems, Tucson, AZ).^30^

### 2.11 Statistical analysis

All data are expressed as the mean ± standard error of the mean (SEM) or the mean ± standard deviation (SD). Statistical analysis was performed using a one‐way repeated‐measures ANOVA, two‐way repeated‐measures ANOVA, Kruskal‐Wallis test, Tukey test, or an unpaired *t*‐test as appropriate. A value of *P *<* *0.05 was considered to be a statistically significant difference.

## 3 RESULTS

### 3.1 In vitro cytotoxicity assay

**Figure 1:**
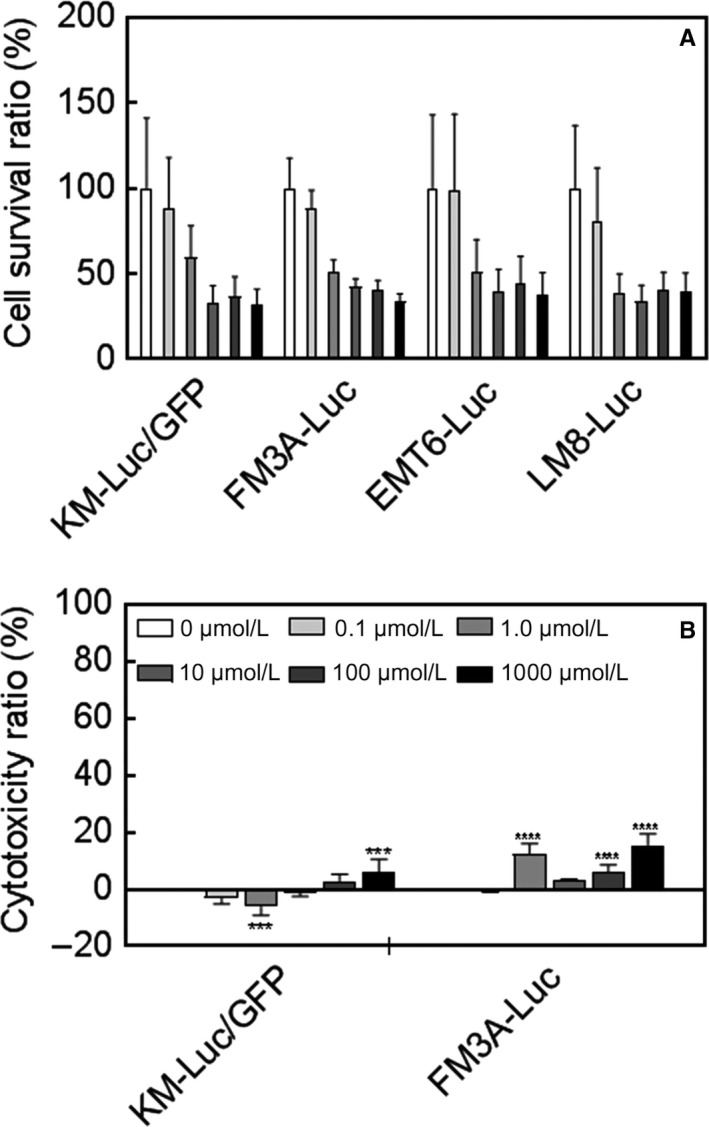
In vitro cytotoxicity and cell survival in the presence of 5‐fluorouracil (5‐FU). (A) Cell survival ratio determined by the MTT assay. 5‐Fluorouracil elicited a concentration‐dependent reduction in cell viability in all cell lines. In KM‐Luc/GFP cells, a statistically significant decrease in cell viability was observed for 1 μmol/L, 10 μmol/L, 100 μmol/L, and 1000 μmol/L 5‐FU (***P <* *0.001, 10 μmol/L vs 0 μmol/L, 100 μmol/L vs 0 μmol/L, and 1000 μmol/L vs 0 μmol/L; *P <* *0.05, 1 μmol/L vs 0 μmol/L; one‐way ANOVA and Dunnett's test). In FM3A‐Luc cells, a statistically significant decrease in cell viability was observed for all concentrations of 5‐FU tested (***P <* *0.001, 1 μmol/L vs 0 μmol/L, 10 μmol/L vs 0 μmol/L, 100 μmol/L vs 0 μmol/L, and 1000 μmol/L vs 0 μmol/L; *P <* *0.05, 0.1 μmol/L vs 0 μmol/L; one‐way ANOVA and Dunnett's test). In EMT6‐Luc cells, statistically significant decreases in cell viability were observed for 1 μmol/L, 10 μmol/L, 100 μmol/L, and 1000 μmol/L 5‐FU (***P <* *0.001, 1000 μmol/L vs 0 μmol/L; **P <* *0.01, 10 μmol/L vs 0 μmol/L, and 100 μmol/L vs 0 μmol/L; *P <* *0.05, 1 μmol/L vs 0 μmol/L; one‐way ANOVA and Dunnett's test). In LM8‐Luc cells, statistically significant decreases in cell viability were observed for 1 μmol/L, 10 μmol/L, 100 μmol/L, and 1000 μmol/L 5‐FU (***P <* *0.001, 1 μmol/L vs 0 μmol/L, 10 μmol/L vs 0 μmol/L, 100 μmol/L vs 0 μmol/L, and 1000 μmol/L vs 0 μmol/L; one‐way ANOVA and Dunnett's test). For each well, the MTT assay was repeated three times for KM‐Luc/GFP and FM3A‐Luc cells, and twice for EMT6‐Luc and LM8‐Luc cells. Data are presented as the mean ± SD (number of wells: KM‐Luc/GFP, n* *= 12; FM3A‐Luc, n =* *12; EMT6‐Luc, n =* *7; LM8‐Luc, n =* *8). (B) Cytotoxicity measured using the LDH assay. 5‐Fluorouracil had only weak cytotoxic actions in KM‐Luc/GFP and FM3A‐Luc cells. In KM‐Luc/GFP cells, a statistically significant increase in the cytotoxicity ratio was observed for 1000 μmol/L 5‐FU (***P <* *0.001, 1000 μmol/L vs 0 μmol/L; one‐way ANOVA and Dunnett's test). In FM3A‐Luc cells, a statistically significant increase in the cytotoxicity ratio was observed for 1 μmol/L, 100 μmol/L, and 1000 μmol/L 5‐FU (***P <* *0.001, 1 μmol/L vs 0 μmol/L, 100 μmol/L vs 0 μmol/L, and 1000 μmol/L vs 0 μmol/L; one‐way ANOVA and Dunnett's test). The LDH assay was performed in triplicate for each experiment (ie, each well). Data are presented as the mean ± SD (number of wells: KM‐Luc/GFP, n =* *8; FM3A‐Luc, n* *=* *9)

The measurement of the cell survival ratio by the MTT assay demonstrated that 5‐FU caused a concentration‐dependent decrease in the viability of all four tumor cell lines (Figure 1A). For KM‐Luc/GFP cells, a statistically significant decrease in cell viability was observed at 1 μmol/L, 10 μmol/L, 100 μmol/L, and 1000 μmol/L 5‐FU (*P *<* *0.001, 10 μmol/L vs 0 μmol/L, 100 μmol/L vs 0 μmol/L, and 1000 μmol/L vs 0 μmol/L; *P *<* *0.05, 1 μmol/L vs 0 μmol/L; one‐way ANOVA and Dunnett's test). For FM3A‐Luc cells, statistically significant decreases in the cell survival ratio were observed at all concentrations of 5‐FU (*P *<* *0.001, 1 μmol/L vs 0 μmol/L, 10 μmol/L vs 0 μmol/L, 100 μmol/L vs 0 μmol/L and 1000 μmol/L vs 0 μmol/L; *P *<* *0.05, 0.1 μmol/L vs 0 μmol/L; one‐way ANOVA and Dunnett's test). In EMT6‐Luc cells, 5‐FU concentrations of 1 μmol/L, 10 μmol/L, 100 μmol/L, and 1000 μmol/L elicited statistically significant decreases in cell viability (*P *<* *0.001, 1000 μmol/L vs 0 μmol/L; *P *<* *0.01, 10 μmol/L vs 0 μmol/L and 100 μmol/L vs 0 μmol/L; *P *<* *0.05, 1 μmol/L vs 0 μmol/L). In LM8‐Luc cells, statistically significant decreases in cell viability were observed at 1 μmol/L, 10 μmol/L, 100 μmol/L, and 1000 μmol/L 5‐FU (*P *<* *0.001, 1 μmol/L vs 0 μmol/L, 10 μmol/L vs 0 μmol/L, 100 μmol/L vs 0 μmol/L, and 1000 μmol/L vs 0 μmol/L).

Since KM‐Luc/GFP and FM3A‐Luc cells have been confirmed to engraft in mice in previous research, these two cell lines were chosen for further experiments. The LDH assay revealed that 5‐FU exerted only minor cytotoxic effects on KM‐Luc/GFP and FM3A‐Luc cells (Figure 1B). For KM‐Luc/GFP cells, a statistically significant increase in the cytotoxicity ratio was observed for 1000 μmol/L 5‐FU (*P *<* *0.001, 1000 μmol/L vs 0 μmol/L; one‐way ANOVA and Dunnett's test). In FM3A‐Luc cells, a statistically significant increase in the cytotoxicity ratio was observed for 1 μmol/L, 100 μmol/L, and 1000 μmol/L 5‐FU (*P *<* *0.001, 1 μmol/L vs 0 μmol/L, 100 μmol/L vs 0 μmol/L, and 1000 μmol/L vs 0 μmol/L; one‐way ANOVA and Dunnett's test).

### 3.2 Measurements of PALN volume before and after the occurrence of metastasis

**Figure 2:**
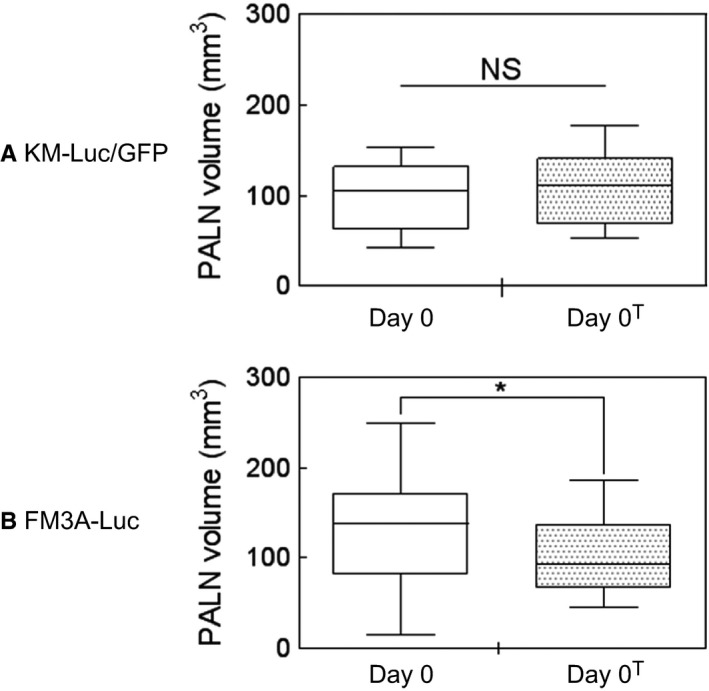
PALN volume before and after the induction of metastasis. (A) PALN volume before the induction of metastasis on day 0 (–6^T^, n =* *13) and after the induction of metastasis with KM‐Luc/GFP cells on day 0^T^ (n* *=* *13). There was no significant change in PALN volume between day 0 and day 0^T^ (NS: not significant; paired *t* test). Data are presented as the mean ± SD. (B) PALN volume prior to metastasis on day 0 (n* *=* *26) and after the induction of metastasis with FM3A‐Luc cells on day 0^T^ (n* *=* *26). A significant decrease in the PALN volume was observed on day 0^T^ compared with day 0 (*P <* *0.05, day 0 vs day 0^T^; paired *t* test). Data are presented as the mean ± SD

To confirm that the present animal model was suitable for the study of false‐negative metastatic LNs, a high‐frequency ultrasound imaging system was used to compare PALN volumes before (day 0) and after (day 0^T^) the induction of metastasis. Figure 2A shows that there was no significant increase in PALN volume between day 0 (–6^T^, *n *=* *13) and day 0^T^ (*n *=* *13) when KM‐Luc/GFP cells were used to induce metastasis. As shown in Figure 2B, PALN volume decreased significantly between day 0 (*n *=* *26) and day 0^T^ (*n *=* *26) when FM3A‐Luc cells were used to induce metastasis (*P *<* *0.05, day 0 vs day 0^T^; paired *t* test). The absence of an increase in PALN volume after the induction of metastasis with KM‐Luc/GFP or FM3A‐Luc cells confirmed that, in both cases, the PALN was a false‐negative metastatic LN.

### 3.3 Treatment of a false‐negative metastatic LN using 5‐FU administered by a LDDS

**Figure 3:**
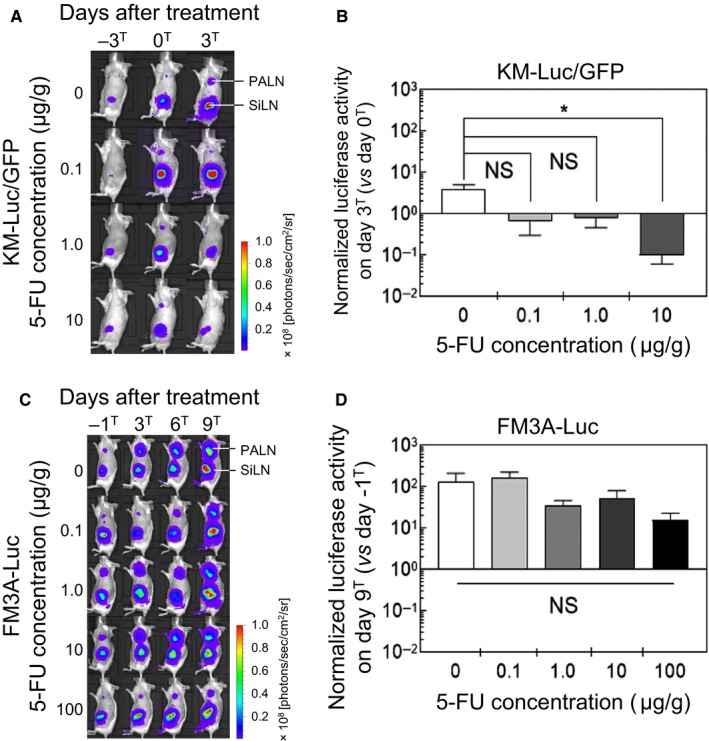
In vivo bioluminescence imaging of luciferase activity before and after the administration of 5‐fluorouracil (5‐FU) using a lymphatic drug delivery system (LDDS). (A) Representative images showing luciferase activity of KM‐Luc/GFP cells in the mouse model of LN metastasis. Luciferase activity was measured on days –3^T^, 0^T^, and 3^T^. (B) Luciferase activity on day 3^T^ normalized to that on day 0^T^. Mice administered 10 μg/g 5‐FU exhibited a significant decrease in luciferase activity when compared with controls (*P <* *0.05, 10 μg/g vs 0 μg/g; one‐way ANOVA and Kruskal‐Wallis test). Although there were trends toward a decrease in luciferase activity in the groups treated with 0.1 μg/g and 1 μg/g 5‐FU, statistical significance was not attained (NS: not significant). Data are presented as the mean ± SD (n =* *4 for each group). (C) Representative images showing luciferase activity of FM3A‐Luc cells in the mouse model of LN metastasis. Luciferase activity was measured on days ‐1^T^, 3^T^, 6^T^, and 9^T^. (D) Luciferase activity on day 9^T^ normalized to that on day ‐1^T^. Compared with the control group, there were no significant decreases in luciferase activity for any of the 5‐FU concentrations tested (NS: not significant; one‐way ANOVA and Kruskal‐Wallis test). Data are presented as the mean ± SD (0 μg/g, n =* *6; 0.1 μg/g, n =* *5; 1 μg/g, n =* *5; 10 μg/g, n =* *5; 100 μg/g, n =* *5)

Luciferase activity was measured to evaluate tumor size before and after the administration of 5‐FU with a LDDS. Luciferase activity was determined on days –3^T^, 0^T^, and 3^T^ for KM‐Luc/GFP cells (Figure 3A) and days –1^T^, 3^T^, 6^T^, and 9^T^ for FM3A‐Luc cells (Figure 3C). Figure 3B shows the luciferase activity of KM‐Luc/GFP cells on day 3^T^ normalized to that on day 0^T^, and Figure 3D shows the luciferase activity of FM3A‐Luc cells on day 9^T^ normalized to that on day –1^T^. When KM‐Luc/GFP cells were used to induce metastasis, administration of 10 μg/g 5‐FU resulted in a significant decrease in luciferase activity compared with the control group (*P *<* *0.05, 10 μg/g vs 0 μg/g; one‐way ANOVA and Kruskal‐Wallis test). There was also a tendency toward a decrease in luciferase activity after the delivery of 0.1 μg/g or 1 μg/g 5‐FU, although statistical significance was not attained (Figure 3B). When FM3A‐Luc cells were used, there were no significant reductions in luciferase activity for any of the 5‐FU concentrations tested (Figure 3D).

### 3.4 Changes in the volumes of the PALN and AALN after 5‐FU administration with a LDDS

**Figure 4:**
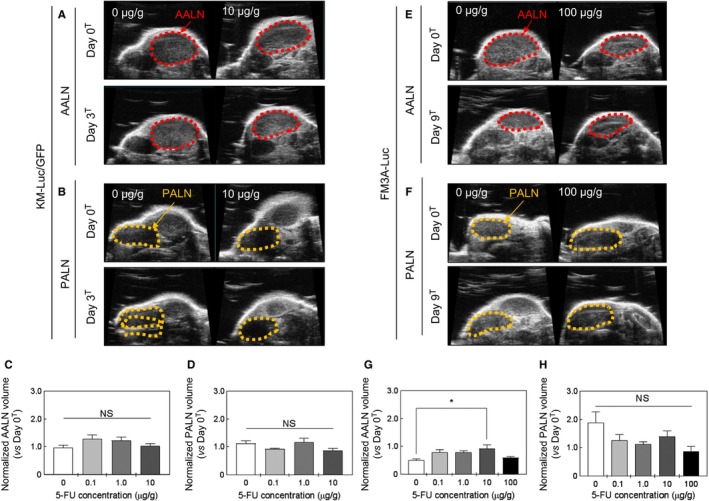
Changes in the volume of the proper axillary lymph node (PALN) and accessory axillary lymph node (AALN) after 5‐fluorouracil (5‐FU) administration with a lymphatic drug delivery system (LDDS). A, B, C, D: KM‐Luc/GFP cells. E, F, G, H: FM3A‐Luc cells. A, Representative 2D ultrasound images of the AALN on day 0^T^ and day 3^T^. B, Representative 2D ultrasound images of the PALN on day 0^T^ and day 3^T^. C, AALN volume normalized to that on day 0^T^. There were no significant differences between groups (0 μg/g, 0.1 μg/g, 1 μg/g, and 10 μg/g 5‐FU; NS: not significant; one‐way ANOVA and Kruskal‐Wallis test). D, PALN volume normalized to that on day 0^T^. There were no significant differences between groups (0 μg/g, 0.1 μg/g, 1 μg/g, and 10 μg/g 5‐FU; NS: not significant; one‐way ANOVA and Kruskal‐Wallis test). Data are presented as the mean ± SEM (0 μg/g, n =* *3; 0.1 μg/g, n =* *3; 1 μg/g, n =* *4; 10 μg/g, n =* *3). E, Representative 2D ultrasound images of the AALN on day 0^T^ and day 9^T^. F, Representative 2D ultrasound images of the PALN on day 0^T^ and day 9^T^. G, AALN volume normalized to that on day 0^T^. AALN volume was significantly greater in the 10 μg/g 5‐FU group than in the control group (*P <* *0.05, 10 μg/g vs 0 μg/g; one‐way ANOVA and Kruskal‐Wallis test). H, PALN volume normalized to that on day 0^T^. There were no significant differences between groups (0 μg/g, 0.1 μg/g, 1 μg/g, and 10 μg/g 5‐FU; NS: not significant; one‐way ANOVA and Kruskal‐Wallis test). Data are presented as the mean ± SEM (0 μg/g, n =* *6; 0.1 μg/g, n =* *5; 1 μg/g, n =* *5; 10 μg/g, n =* *5; 100 μg/g, n =* *5)

Changes in AALN and PALN volumes (assessed using a high‐frequency ultrasound imaging system) were used to evaluate the antitumor effect of 5‐FU. Figure 4A‐D show results for experiments using KM‐Luc/GFP cells. Figure 4A and B show representative images of the AALN and PALN on day 0^T^ and day 3^T^ for 5‐FU concentrations of 0 μg/g (control) and 10 μg/g. Figure 4C and D show AALN and PALN volumes, respectively, normalized to the volume on day 0^T^: there were no significant differences between the groups. Figure 4E‐H present data for experiments that used FM3A‐Luc cells. Figures 4E and F show representative images of the AALN and PALN on day 0^T^ and day 9^T^ for 5‐FU concentrations of 0 μg/g (control) and 100 μg/g. Figure 4G and H shown AALN and PALN volumes normalized to the volume on day 0^T^. AALN volume was significantly greater in the 10 μg/g group than in the control group (*P *<* *0.05, 10 μg/g vs 0 μg/g; one‐way ANOVA and Kruskal‐Wallis test), although no significant changes in AALN volumes were observed for other concentrations of 5‐FU (Figure 4G). There were no significant differences in the PALN volume between groups (Figure 4H).

### 3.5 Ex vivo assessment of the antitumor effect of 5‐FU

**Figure 5:**
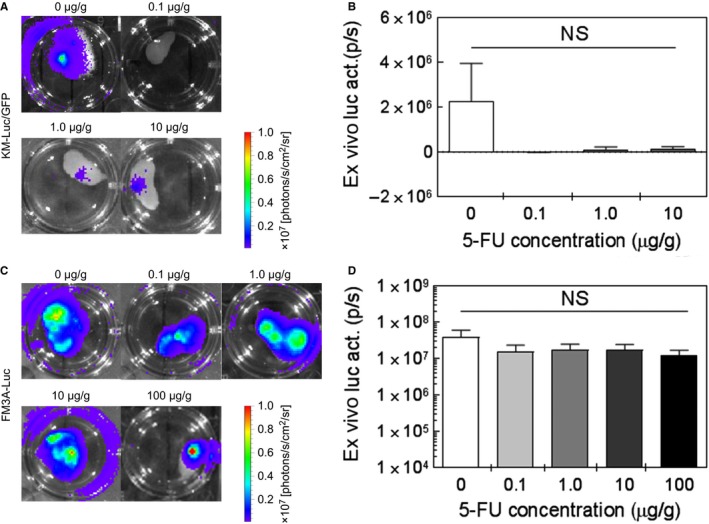
*Ex vivo* assessment of the antitumor effect of 5‐fluorouracil (5‐FU). A, B: KM‐Luc/GFP cells. C, D: FM3A‐Luc cells. A, C: Representative ex vivo bioluminescence images of the proper axillary lymph node (PALN) for each group (ie, each concentration of 5‐FU) on day 3^T^ for KM‐Luc/GFP cells (A) and day 9^T^ for FM3A‐Luc cells (C). B, D: Ex vivo bioluminescence intensity for KM‐Luc/GFP cells (B) and FM3A‐Luc cells (D). There were no significant differences in ex vivo bioluminescence intensity between the control group and each 5‐FU group for both KM‐Luc/GFP cells and FM3A‐Luc cells (NS: not significant; one‐way ANOVA and Kruskal‐Wallis test). Data are presented as the mean ± SEM (0 μg/g, n =* *6; 0.1 μg/g, n =* *5; 1 μg/g, n =* *5; 10 μg/g, n =* *5; 100 μg/g, n =* *5)

Figure 5 shows representative ex vivo bioluminescence images of the PALN for each group (ie, each 5‐FU concentration). In vivo bioluminescence intensity was measured on day 3^T^ for KM‐Luc/GFP cells and day 9^T^ for FM3A‐Luc cells. The PALN was then excised to permit measurements of ex vivo bioluminescence intensity. There were no significant differences in ex vivo bioluminescence intensity between the control group and each 5‐FU group for both KM‐Luc/GFP cells (Figure 5A and B; one‐way ANOVA and Kruskal‐Wallis test) and FM3A‐Luc cells (Figure 5C and D; one‐way ANOVA and Kruskal‐Wallis test).

### 3.6 Blood biochemistry and animal weight

To determine whether the use of the LDDS was associated with toxicity, blood biochemical parameters on day 3^T^ (KM‐Luc/GFP cells) and day 9^T^ (FM3A‐Luc cells), and changes in animal weight during the study were assessed. There were no significant changes in biochemical parameters or body weight for both cell lines (Table 1).

**Table 1:** Evaluation of the acute toxicity of 5‐FU

	KM‐Luc/GFP			FM3A‐Luc			
	0 μg/g (*n *=* *4)^a^	10 μg/g (*n *=* *4)^a^	Statistical significance^b^	0 μg/g (*n *=* *6)^a^	10 μg/g (*n *=* *5)^a^	100 μg/g (*n *=* *5)^a^	Statistical significance^c^
CRE (mg/dL)	0.10 ± 0.01	0.10 ± 0.01	NS	0.08 ± 0.02	0.09 ± 0.01	0.09 ± 0.02	NS
BUN (mg/dL)	26.6 ± 3.4	34.4 ± 15.9	NS	34.1 ± 10.3	32.8 ± 9.7	32.5 ± 5.3	NS
T‐BIL (IU/L)	0.04 ± 0.03	0.02 ± 0.01	NS	0.04 ± 0.03	0.13 ± 0.18	0.03 ± 0.02	NS
AMY (IU/L)	2001.0 ± 88.3	2275 ± 741.8	NS	2115.3 ± 391.8	2302.4 ± 647.5	2117.2 ± 180.0	NS
ALT (IU/L)	37.5 ± 6.2	44.3 ± 12.0	NS	51.0 ± 15.1	66.8 ± 43.1	35.2 ± 6.5	NS
AST (IU/L)	127.0 ± 32.3	115.3 ± 19.1	NS	144.2 ± 37.1	157.8 ± 66.2	122.2 ± 21.8	NS
Weight change (g)	−1.00 ± 1.22	−0.25 ± 0.43	NS	−1.00 ± 2.94	1.60 ± 1.36	0.40 ± 0.80	NS

Samples were obtained on day 3^T^ for KM‐Luc/GFP cells and on day 9^T^ for FM3A‐Luc cells.

CRE, creatinine; BUN, blood urea nitrogen; T‐BIL, total bilirubin; AMY, amylase ; ALT, alanine aminotransferase; AST, aspartate aminotransferase; NS, not significant.

a Values are mean ± SEM.

b Unpaired *t* test.

c Kruskal‐Wallis test.

### 3.7 Histological analysis of the PALN

**Figure 6:**
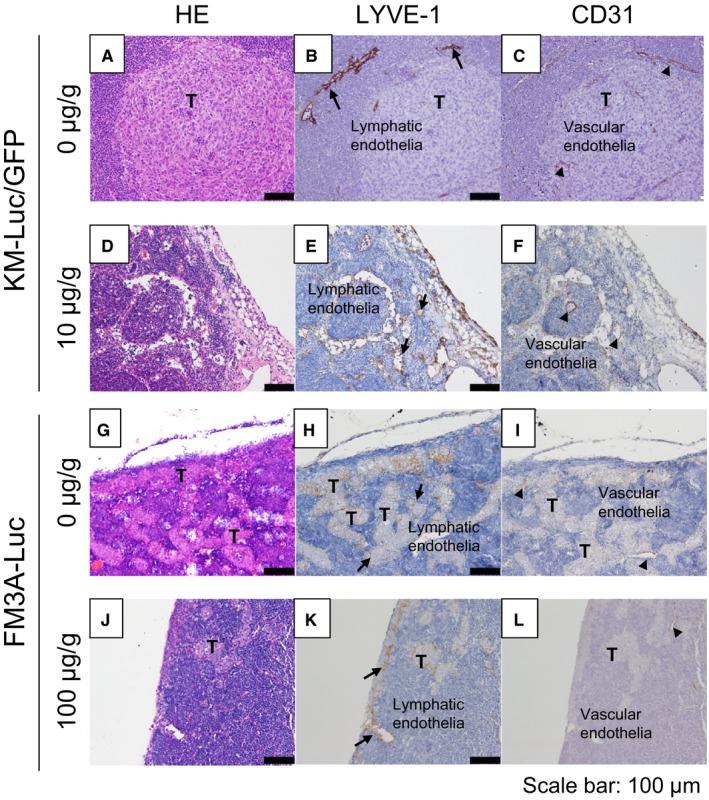
Histological analysis of the proper axillary lymph node (PALN). A‐F: KM‐Luc/GFP cells. In the control group (A‐C), tumor cells were detected in and near the marginal sinus and in the vicinity of the lymphatic sinus (arrows) and blood vessels (arrow heads). In the 10 μg/g 5‐fluorouracil (5‐FU) group (D‐F), a region containing tumor cells was not detected. G‐L: FM3A‐Luc cells. In both the control group (G‐I) and 10 μg/g 5‐FU group (J‐L), tumor was detected in the lymphatic sinus (H, K) but not in blood vessels (I, L). A, D, G, J: HE staining. B, E, H, K: anti‐LYVE‐1 staining. C, F, I, L: anti‐CD31 staining. T: tumor. Scale bar: 100 μm

Figure 6 shows histological analysis of the PALN after the administration of 5‐FU with the LDDS. In experiments using KM‐Luc/GFP cells, tumor cells were detected in and near the marginal sinus in the control group (Figure 6A‐C), with tumor cells located near the lymphatic sinus and blood vessels (arrows in Figure 6B and C). In the 10 μg/g 5‐FU group, tumor‐containing regions were not detected (Figure 6D‐F). In experiments utilizing FM3A‐Luc cells, a tumor‐containing region was detected in the lymphatic sinus (Figure 6H and K) but not blood vessels (Figure 6I and L) in both the groups.

## 4 DISCUSSION

Lymph node metastasis occurs in many carcinomas, and its treatment improves the prognosis of patients. Lymph node metastasis has been thought to result from tumor cells breaking off from the primary tumor to enter the lymphatic vessels and pass through a series of LNs before reaching the thoracic duct and subclavian vein.^31^ Recently, the theory of LN‐mediated hematogenous metastasis was advocated,^6^, ^9^ suggesting that tumor cell invasion from the marginal sinus into extranodal veins during early stage LN metastasis could be the starting point for hematogenous metastasis.^6^ In other words, systemic metastasis could potentially be prevented by treating false‐negative metastatic LNs. Furthermore, there is evidence that dormant cancer cells may be activated by surgical dissection of LNs in patients^32^, ^33^ and in a mouse model.^34^, ^35^, ^36^ These findings question whether conventional LN dissection contributes substantially to an improvement in the prognosis of patients.

The present study first explored the cytotoxic effects of 5‐FU in vitro and then evaluated the therapeutic effects of 5‐FU administered by a LDDS in a mouse model of a false‐negative metastatic LN, which was developed using two different tumor cell lines (KM‐Luc/GFP and FM3A‐Luc cells). In vitro experiments revealed that four different cell lines were sensitive to increasing concentrations of 5‐FU (Figure 1). In vivo experiments in MXH10/Mo/lpr mice demonstrated a significant antitumor effect of 5‐FU against KM‐Luc/GFP cells but not FM3A‐Luc cells (Figures 3, 4, 5, 6). This discrepancy may reflect differences in the invasion patterns of the two cell types. KM‐Luc/GFP cells have low invasive growth characteristics and form tumor regions with well‐defined borders in or near the marginal sinuses (Figs 6A‐D).^24^ On the other hand, FM3A‐Luc cells have high invasive growth characteristics, proliferate along the trabecular sinus, and invade the cortex and paracortex (Figs 6I‐P).^37^ Drugs delivered from the AALN to the PALN likely reached KM‐Luc/GFP cells proliferating in the marginal sinus but not FM3A‐Luc cells invading the trabecular sinus and cortex. Under normal physiological conditions, about 90% of lymph flows into a peripheral path via the marginal and medullary sinuses,^38^ that is, drugs in the marginal and medullary sinuses are washed out by lymph.^39^ For this reason, a LDDS needs to deliver drugs into the medulla of a metastatic LN. Although physicochemical properties such as particle size, composition, dose, surface charge, and molecular weight are considered to be important factors determining the in vivo behavior of particulates, another factor facilitating the delivery of a drug to a downstream LN is the rate of drug injection into an upstream LN. We found that intranodal injection of a drug into an upstream LN at a rate < 100 μL/min allowed downstream LNs to accumulate the drug^40^ and this efficacy was explained by the impulse values calculated from injection pressures in the upstream LN.

The present study used MXH10/Mo/lpr mice, which show systemic lymphadenopathy from 8 weeks of age due to the accumulation of lpr‐T cells.^41^ MXH10/Mo/lpr mice do not express the *fas* gene involved in apoptosis, since the *lpr* gene is a *fas*‐deletion mutant gene. Thus, the immune system in MXH10/Mo/lpr mice is functional except for the signaling pathway related to *fas*. Most previous studies of LN metastasis have used experimental systems based on human xenografts formed in immunocompromised mice. Given the relevance of immune function to LN metastasis, we consider MHX10/Mo/lpr mice to be a superior model system to immunocompromised mice.

The present study examined the effects of 5‐FU, which inhibits DNA synthesis by restricting the availability of thymidylate during the S phase of the cell cycle.^42^ Our previous investigations utilized cisplatin, a platinum‐containing compound belonging to the alkylating agent family,^23^, ^43^, ^44^ and doxorubicin, which inhibits the progression of an enzyme (topoisomerase II) that relaxes DNA supercoils involved in transcription.^26^ Both cisplatin and doxorubicin were effective in the treatment of metastatic LNs when administered using a LDDS, indicating that conventional drugs used in the clinic have the potential to treat metastatic LNs when administered with this novel approach. A potential important advantage of a LDDS over systemic chemotherapy is that treatment of a metastatic LN requires much smaller quantities of drug (1/100 to 1/10 000 of the amount), which would minimize side effects due to drug administration (Table 1).^23^, ^26^, ^43^, ^44^ This finding strongly indicates that drug administration with a LDDS would likely not be associated with serious adverse events. We anticipate that the effectiveness of drug administration with a LDDS in the treatment and prevention of false‐negative metastatic LNs will be evaluated further in future clinical trials.

## CONFLICTS OF INTEREST

Tetsuya Kodama received commercial research support from Yakult Honsha Co., Ltd. The other authors declare no conflicts of interest.
